# The effects of climatic fluctuations and extreme events on running water ecosystems

**DOI:** 10.1098/rstb.2015.0274

**Published:** 2016-05-19

**Authors:** Guy Woodward, Núria Bonada, Lee E. Brown, Russell G. Death, Isabelle Durance, Clare Gray, Sally Hladyz, Mark E. Ledger, Alexander M. Milner, Steve J. Ormerod, Ross M. Thompson, Samraat Pawar

**Affiliations:** 1 Department of Life Sciences, Imperial College London, Silwood Park Campus, Ascot, Berkshire SL5 7PY, UK; 2 Group de Recerca Freshwater Ecology and Management (FEM), Departament d'Ecologia, Facultat de Biologia, Institut de Recerca de la Biodiversitat (IRBio), Universitat de Barcelona (UB), Diagonal 643, Barcelona, Catalonia, 08028 Spain; 3 School of Geography and Water, University of Leeds, Woodhouse Lane, Leeds LS2 9JT, UK; 4 Institute of Agriculture and Environment—Ecology, Massey University, Private Bag 11-222, Palmerston North 4442, New Zealand; 5 Water Research Institute and Cardiff School of Biosciences, Cardiff CF10 3AX, UK; 6 School of Biological Sciences, Monash University, Clayton, Melbourne, Victoria 3800, Australia; 7 School of Geography, Earth and Environmental Sciences, University of Birmingham, Edgbaston, Birmingham B15 2TT, UK; 8 Institute of Arctic Biology, University of Alaska Fairbanks, Fairbanks, AK 99775, USA; 9 Institute for Applied Ecology, University of Canberra, Australian Capital Territory 2601, Australia; 10 School of Biological and Chemical Sciences, Queen Mary University of London, London E1 4NS, UK

**Keywords:** ecosystem functioning, biodiversity, metabolism, community assembly, food webs, resilience

## Abstract

Most research on the effects of environmental change in freshwaters has focused on incremental changes in average conditions, rather than fluctuations or extreme events such as heatwaves, cold snaps, droughts, floods or wildfires, which may have even more profound consequences. Such events are commonly predicted to increase in frequency, intensity and duration with global climate change, with many systems being exposed to conditions with no recent historical precedent. We propose a mechanistic framework for predicting potential impacts of environmental fluctuations on running-water ecosystems by scaling up effects of fluctuations from individuals to entire ecosystems. This framework requires integration of four key components: effects of the environment on individual metabolism, metabolic and biomechanical constraints on fluctuating species interactions, assembly dynamics of local food webs, and mapping the dynamics of the meta-community onto ecosystem function. We illustrate the framework by developing a mathematical model of environmental fluctuations on dynamically assembling food webs. We highlight (currently limited) empirical evidence for emerging insights and theoretical predictions. For example, widely supported predictions about the effects of environmental fluctuations are: high vulnerability of species with high *per capita* metabolic demands such as large-bodied ones at the top of food webs; simplification of food web network structure and impaired energetic transfer efficiency; and reduced resilience and top-down relative to bottom-up regulation of food web and ecosystem processes. We conclude by identifying key questions and challenges that need to be addressed to develop more accurate and predictive bio-assessments of the effects of fluctuations, and implications of fluctuations for management practices in an increasingly uncertain world.

## 1. Introduction

Although climate change is a natural part of the Earth system, the rates predicted over the next century far exceed those of recent decades [1,2]. A common prediction is for a general trend of future warming overlain with increasingly frequent and more intense fluctuations and extreme events [1,2]. Examples of the latter include meteorological events such as heatwaves and physical phenomena such as floods. Such environmental fluctuations can have profound effects on freshwater ecosystems, yet they are rarely studied via explicit integration of theoretical and empirical approaches [3]. Running waters supply many ecosystem goods and services that are especially vulnerable to climate change (e.g. flood prevention, potable water and irrigation for agriculture), because they are relatively small and fragmented in the landscape [1,2,4].

Although we have ever-more sophisticated climatic models to project future changes, the effects of climatic fluctuations on multi-species, complex ecosystems remain poorly understood, both empirically and theoretically [5,6], over 40 years after the first theoretical explorations of how environmental stochasticity affects food web dynamics [7,8]. The problem is empirical as much as theoretical—for example, the apparently general theoretical prediction that environmental fluctuations shorten food chains remains largely untested (but see [6,9,10]). The effects of environmental fluctuations, through food web dynamics, on ecosystem properties, are even less well understood, especially in running waters, which are inherently dynamic, both physically and biologically [11–15]. For example, running water ecosystems are constantly perturbed by changes in catchment geomorphology and land-use, local physico-chemical parameters, and changes in timings of extreme events relative to the normal seasonal cycle [16,17]. Furthermore, although extreme events may be viewed simply as one end of a gradient of fluctuations, anthropogenic influences are increasingly altering their intensity, frequency and duration, with potentially dramatic consequences for running waters [15]. Separating the biological effects of extreme events from the effects of inherent and chronic background fluctuations in running waters is an additional and important challenge.

Indeed, the susceptibility of target species will depend upon the context, magnitude, extent and timing of environmental fluctuations: extreme climatic events do not always have extreme ecological consequences, and multiple events (e.g. a wildfire followed by a flood) that are not necessarily individually extreme can have extreme biological impacts when combined [18,19]. In addition, climatic fluctuations are themselves often components of a general underlying (e.g. warming) trend, in which both average conditions and variability change over time. Jentsch *et al.* [20] refer to ‘trend effects’ and ‘event effects’, with the latter often superimposed over the former [15,21].

Here, we propose a new mechanistic framework for tackling the challenge of developing a better, more predictive understanding of the effects of environmental fluctuations and extreme events on running-water ecosystems. This necessarily involves linking theoretical and empirical research from individual metabolism and biomechanics to whole ecosystems, as well as a consideration of thresholds of disturbance that change key food web and ecosystem properties.

**Figure 1.**
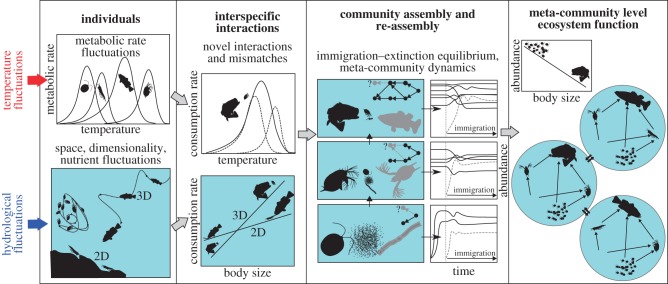
A conceptual framework for studying the effects of environmental fluctuations on running water ecosystems. ‘2D’ and ‘3D’ refer to two and three spatial dimensions, respectively. Both, temperature and hydrological fluctuations affect individuals, which are then propagated through species interactions to higher levels of organization. Species interactions are likely to be disrupted also due to inter-specific mismatches arising from the differing tolerances, physiologies or biomechanics of predator and prey. Note also that ectotherm thermal performance curves are typically asymmetric (as shown)—i.e. heat waves are likely to have far stronger impacts than cold spells on species and interactions.

We mainly focus on temperature and hydrological fluctuations as the sources of environmental fluctuations associated with climate change for two reasons. First, effects of climatic fluctuations are primarily manifested in running waters via abnormal temperature and/or hydrological regimes [15,22]. Second, these perturbations have mechanistically understandable and predictable effects on individual physiology (e.g. increasing metabolic rate with warming; [23,24]) and species interactions (e.g. increased predator–prey encounter rates in hydrological refugia [25–27]). Indeed, we argue that effects of environmental fluctuations will remain difficult to predict unless we focus more carefully on mechanisms at lower levels of organization (individuals, interactions; figure 1), because this is where fluctuations in temperature and hydrology will have relatively predictable consequences.

The timing or characteristic frequency of environmental fluctuations is also important, as it determines the severity of disruptions to the natural phenology of rates and traits within species (e.g. oviposition, emergence, pupation, diapause) as well as species interactions [28,29]. Our framework therefore requires the combination of metabolic constraints and timescales with the temporal scaling of the relevant perturbations.

The type of event and connectivity within and among running-water ecosystems also determines population recruitment and the buffering capacity of refugia. For example, floods expand and homogenize riverine habitats (e.g. [17]), whereas droughts constrain and fragment them (e.g. [30]). This has important implications for the recovery or assembly of local communities by the re-establishment of existing interactions as well as stabilization of novel interactions over time: studying how fluctuations affect dynamically assembling ecosystems is therefore an integral part of our framework.

To illustrate our framework, we develop a mathematical model that maps environmental fluctuations, as well as extreme events, onto individual populations in dynamically assembling food webs. The results demonstrate how food webs might respond to increasing intensity of disturbances, and provide both heuristic and testable predictions, many of which are broadly consistent with the currently available (but still limited) empirical evidence. Our framework and preliminary theoretical explorations also highlight the potential for developing more objective ways of defining ‘extreme events’ based upon the changes they bring about in ecosystem properties, and for disentangling the effects of chronic fluctuations from those of extreme events in running waters.

In the following sections, we outline our conceptual and theoretical framework for studying the effects of fluctuations and extreme events on ecosystems, and develop a stochastic food web model that illustrates this framework. We then consider data and case studies that both summarize our current empirical knowledge about effects of fluctuations, and provide evidence (or lack thereof) for some of the key predictions of the model. Finally, we review current understanding of the effects of environmental fluctuations on ecosystem services and implications for potential management and mitigation strategies to cope with future extreme events, and end by identifying a suite of potential new empirical and theoretical avenues for research.

## 2. A conceptual and theoretical framework

We propose that developing a mechanistic understanding for running-water ecosystems requires empirical data and theoretical models for four key components (figure 1): (i) effects of fluctuations on individual metabolism and biomechanics; (ii) effects of fluctuations on species interactions; (iii) community assembly/re-assembly dynamics; and (iv) quantification of ecosystem functioning. We argue that these components are key to understanding how fluctuations and extreme events can have direct effects on individual fitness and population abundance as well as indirect effects that propagate through the food web [27,31,32]. These components and how they integrate are illustrated in figure 1.

### (a) Setting the scene: effects of environmental fluctuations on individual metabolism and performance traits

Environmental fluctuations first and foremost affect individual metabolism, which then determines performance traits (e.g. movement and dispersal through the landscape) [23,24,27,33]. A simple yet powerful model that captures dominant, inherent constraints on whole-individual metabolic rate *P* (J s^–1^) is
2.1
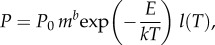
where *P*_0_ is a taxon- and metabolic state-dependent normalization constant; *m* is body mass (kg); *b* is a scaling exponent (dimensionless); *E* is thermal sensitivity and includes the emergent effect of the activation energies (eV) of rate-limiting steps in underlying biochemical reactions (1 eV = 96.49 kJ mol^–1^); *k* is the Boltzmann constant (8.62 × 10^–5^ eV K^–1^); *T* is body temperature (in kelvin); and *l*(*T*) is a function that captures the decrease in metabolic rates at higher-than-optimal temperatures [23,27,34], relevant when thermal fluctuations are extreme. The size-scaling component of metabolic rate in equation (2.1), when measured across species, is allometric with *b* ≈ 0.75 for multicellular eukaryotes, but may be more variable across other domains of life (e.g. *b* > 0.75 or even > 1 in unicellular protists and prokaryotes) [24,35,36]. This scaling of metabolic rate with body size is a primary reason why size is such a good proxy for a wide range of organism- and population-level properties, from fecundity and dispersal ability to trophic position and population density, all of which determine organismal and population resistance or resilience to extreme events. Body size is also important because it strongly determines an individual's effective temporal and spatial scales of operation (e.g. generation times scale positively and intrinsic growth rates scale negatively with body size [24,37]), and thus determines which temporal and spatial scale of fluctuations have the strongest effect.

We emphasize that equation (2.1) captures only the *dominant inherent* constraints of body size and temperature on the metabolic rate of an individual organism. Indeed, equation (2.1) has often been used to parametrize models of ecosystems in stable environments [28,37–40]. However, as such, it cannot capture the effects of environmental fluctuations other than environment-driven changes in body temperature, *T.* For example, hydrological extremes alter, in both time and space, the Euclidean dimensions within which organisms of particular body sizes interact with the physical medium and with each other. In order to model the effects of environmental fluctuations, such as changes in spatial dimensionality or complexity (figure 1) and nutrient concentration, equation (2.1) necessarily needs to be extended to include the effects of environment-driven fluctuations in the metabolic cost of locomotion or nutrient uptake on metabolic rate, *P*. Tackling this issue is one of the fundamental future challenges in research on running-water ecosystems. Effects of extreme flows may well be quantifiable in a manner analogous to thermal performance curves. The empirical characterization of LIFE scores of invertebrate responses to flows (e.g. relevant to drought or flood conditions) represents an important first step in this direction [41].

### (b) Effects of environmental fluctuations on species interactions

Through the effects of temperature and hydrological fluctuations in running-water systems on individual metabolism and performance, the rate of interactions between species as well as between species and their abiotic resources (at lower trophic levels) can be altered [26,27,42]. For example, hydrological fluctuations often perturb the dynamics of interacting species by depressing population sizes (e.g. through washout during floods). New theoretical and empirical studies are currently developing models analogous to equation (2.1) for pairs of interacting species [26,27,43,44]. These interaction models are necessary for scaling up the effects of metabolic fluctuations to the food web and ecosystem levels of organization (figure 1). A general formulation of *per capita* biomass consumption rate *c* (mass.time^–1^) resulting from a trophic interaction is [27,43]
2.2
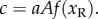


Here, *x*_R_ is resource biomass density (mass area^–1^ or volume^–1^), *a* is search rate (area or volume time^–1^), *A* is probability of attack success (conditional on attack), and *f*(*x*_R_) is the prey risk function that determines the shape of the consumer's functional response. Metabolically and biomechanically constrained equations for the parameters in the right-hand side of equation (2.2) allow both temperature fluctuations and size effects to be mapped onto species interaction rates [26,27,40,43,45].

Considering interactions in a mechanistic way allows a better understanding of how mismatches in physiology or other functional traits between interacting species (e.g. differences in body size or thermal sensitivity, *E*) can either amplify or dampen the effects of fluctuations on consumer–resource and food web dynamics [26,27,46]. Such mismatches are important because hydrological fluctuations in running-water systems often bring new species into contact (e.g. by aggregating in flow refugia, or homogenization of lentic and lotic habitats during floods; [30,47]), and also change the phenology of life history (e.g. egg-laying) and performance traits (e.g. diel activity pattern) [48–50]. Mechanistic models for the components of interaction rate (equation (2.2)) have recently been derived which demonstrate that consumption rate depends upon the differences in body sizes and thermal sensitivities (*E*) of the interacting species [26,27,46], and greater mismatches can destabilize consumer–resource dynamics. Below, we make the first theoretical exploration of the potential effect of such mismatches at the food web level when environments fluctuate (electronic supplementary material, appendix 1 and figure S2).

### (c) Community assembly and re-assembly dynamics

Theory and data on community assembly rates and rules are particularly necessary in running waters, because they are inherently subjected to multiple disturbances over both space and time in addition to those exerted by more extreme climatic events. This requires the study of both ecological resistance (system’s ability to remain unchanged in the face of a perturbation) and resilience (system's ability to rebound or return to a stable state after a perturbation). Theoretically, this involves the study of open, dynamically assembled (and constantly re-assembled) local communities. A general mathematical model that allows the first two components of our framework to be embedded into an *S*-species consumer–resource (including inorganic substrate) food web system is
2.3
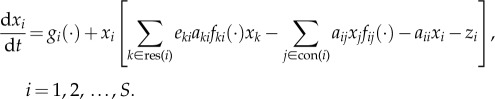


Here, *x_i_* is the *i*th species biomass density (mass area^–1^ or volume^–1^), function *g_i_*(·) is biomass production rate (mass × time^–1^ × area^–1^ or ×volume^–1^), res(*i*) and con(*i*) are sets of its resources and consumers, respectively, *a_ii_* is a coefficient (volume × mass^–1^ × time^–1^) for biomass loss rate due to intraspecific interference, *a_ij_* is the rate at which habitat volume is cleared by consumer *j* (volume × mass^–1^ × time^–1^) (the ‘search rate’), *z*_i_ (time^–1^) is intrinsic (density-independent) biomass loss rate due to respiration, mortality or outflow, *e_ij_* (a proportion) is conversion efficiency of resource to consumer biomass, and *f*(·) (dimensionless) determines the shape of the resource uptake rate function (the functional response). Equation (2.3) is a general model because different specifications of its parameters yield particular models, including Lotka–Volterra (for *g*(·) = *r*_max_ *x*_i_ and *f*(·) = 1) [7,38,51,52], Rosenzweig–MacArthur (*g*(·) = *r*_max_ *x*_i_, *a_ii_* = 0 and *f*(·) = Type II functional response) [43,46,53], ‘bio-energetic’ models (*f*(·) = multi-species functional response) [40,54] or Monod-like (*g*(·) = dilution rate-dependent substrate flux, *a_ii_* = 0 and *f*(·) = saturating uptake function) [55]. Below we use a Lotka–Volterra specification of equation (2.3) to illustrate our framework and make a preliminary theoretical exploration into the effects of fluctuations on running-water ecosystems (electronic supplementary material, appendix S1).

### (d) Quantifying ecosystem functioning

The final component of the mechanistic framework is the challenge of quantifying ecosystem functioning among multiple (potentially intermittently) connected local communities in a running-water landscape. This necessarily requires development of quantitative measures of how ecosystem functioning or services relate to the underlying components of individual metabolism, interactions, mismatches and assembly dynamics (figure 1). For example, gross primary production (GPP) and ecosystem respiration (ER) are key ecosystem functions that determine the capacity of ecosystems to sequester CO_2_. These are empirically measureable at high spatial and temporal resolution [56], but linking ecosystem functioning to the parameters or state variables of models of the underlying levels (equations (2.1)–(2.3)) is a new and exciting challenge that remains largely open. A starting point could be to find simple measures that link net productivity of all autotrophs in the system (equation (2.3)) (effective production minus losses due to respiration e.g., sums of all autotroph level *x_i_*(*a_ii_ x_i_* + *z_i_*) terms in equation (2.3)). The sum of the biomasses across the *S* populations of all organisms (autotrophs + heterotrophs) in the ecosystem may also be another meaningful measure. Our mechanistic framework potentially allows measures of ecosystem functioning to be linked explicitly to particular individual or interaction parameters under a given regime of environmental fluctuations. For example, rates of ecosystem functioning may decline with increasing levels of physiological mismatches between species.

### (e) Putting it together: a mathematical model

We now illustrate how the components of our mechanistic framework can be integrated into a single model of ecosystem dynamics. Because running waters also experience considerable fluctuations in nutrient inputs due to hydrological changes, we consider both climate-driven metabolic fluctuations as well as changes in carrying capacity such as those arising from hydrological fluctuations. The details of the model are given in the electronic supplementary material, appendix S1. As required by our framework, this model explicitly incorporates assembly dynamics, rather than the more traditional stability analyses of fixed-size food web systems (electronic supplementary material, appendix S1). Our modelling stops short of mapping local food web properties onto the ecosystem of such meta-communities (the fourth component, figure 1). This is one of the key areas for future work.

**Figure 2.**
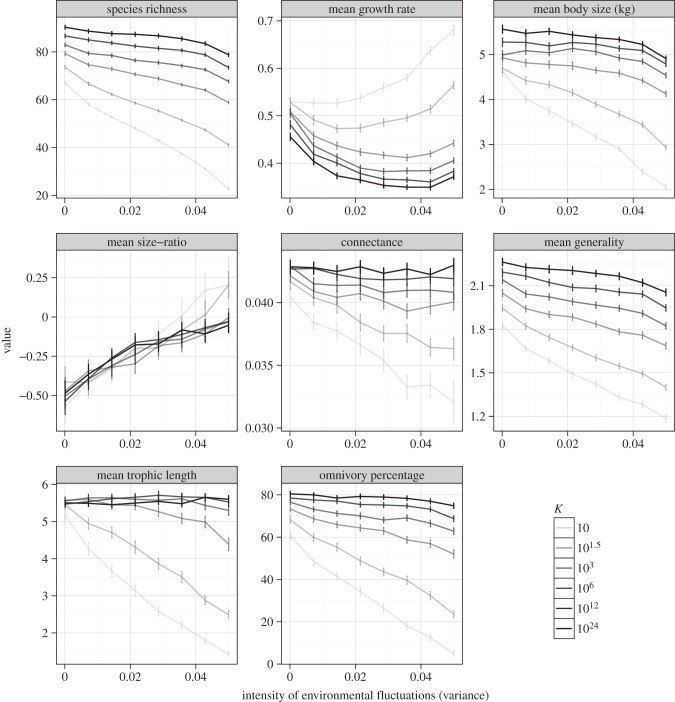
The effect of environmental fluctuations on dynamically assembled model ecosystems. Changes in key food web features in response to increasing intensity of environmental fluctuations (environmental variance, 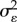; equation S3) are shown at different carrying capacities (*K*). Size-based properties—mean size and consumer–resource size ration—are on log_10_ scale. The bars represent 95% confidence intervals around the mean of 200 community assembly simulations. Each model community at the end of a simulation is at immigration–extinction equilibrium. Model structure and parametrizations are detailed in the electronic supplementary material, appendix S1.

Some key results from the modelling are shown in figure 2 (also see the electronic supplementary material, figures S1–S4), and summarized in the electronic supplementary material, table S1. Several key insights emerge from this model:

(i) Whether a certain regime of fluctuations (the environmental variance *σ*^2^) is extreme (i.e. whether it causes a qualitative change it the system's dynamical behaviour or structural properties) depends upon the carrying capacity. Thus, for example, a hydrological fluctuation resulting in a change in nutrient availability can either amplify or dampen the effects of temperature fluctuations in a predictable way.
(ii) Environmental fluctuations can ‘select’ for particular properties of size distributions and the related intrinsic growth rates of species (because growth rates scale negatively with size).
(iii) Certain ecosystem properties such as species' body distributions change non-monotonically with increasing fluctuations.
(iv) Mismatches or differences among species in how they experience environmental fluctuations can qualitatively change resilience to environmental fluctuations (electronic supplementary material, figure S2), with the results suggesting that ecosystem resilience to fluctuations could be negatively correlated with the level of mismatches between species.

Furthermore, we note that the theoretical framework and the model developed here potentially allow the effects of extreme events to be separated from those of the chronic ‘background’ fluctuations typical of running-water ecosystems. For example, comparing food webs assembled under extreme events without background fluctuations versus those with background chronic perturbations only would be an important step towards disentangling the effects of the two types of perturbations (electronic supplementary material, figure S5). We now consider empirical evidence (or lack thereof) within the context of our integrative framework, and consider various directions in which the framework would need to be extended to capture realistically complex scenarios of fluctuations arising from climatic temperature, hydrology, as well as combinations of these two.

## 3. Current empirical knowledge and links to theoretical predictions

### (a) Thermal fluctuations and extremes

Heatwaves are spikes of abnormally hot weather, and although relatively few studies have explicitly investigated their effects in rivers, experimentally increasing the frequency, intensity and duration of warming can alter the rates of emergence of aquatic insects and community composition [57]. For instance, the 2003 European heatwave caused high mortality among riverine benthic invertebrates in France and major shifts in community structure that lasted for almost a decade [21,58]. During a heatwave, individuals may be pushed outside their optimal envelope within their thermal performance curve (individual level panel in figure 1). Smaller organisms tend to be favoured under warmer conditions ([59], but see [57]), and this should also extend to heat waves due to the allometric scaling of metabolism—and hence many physiological and ecological processes—with body mass. As each individual must meet its metabolic demands, larger organisms will suffer disproportionately under rising temperatures because of their higher *per capita* metabolic rate [24]. The consequences for individuals will ultimately ramify through to the community they comprise, the interactions they have within the food web, and the ecosystem processes they generate en masse. Larger, longer-lived organisms with slower life cycles are most likely to face local extinction, because such acute effects are the strongest when manifested within a single generation, whereas smaller species at the lower trophic levels may benefit from reduced top-down control, particularly if indirect food web effects outweigh the direct metabolic costs. Some of these effects are apparent in our theoretical results, with size distributions of dynamically assembled food webs in fluctuating environments tending towards smaller species on average, and become more skewed towards smaller organisms (figure 2; electronic supplementary material, figure S3 and table S1).

Different ecosystem processes have distinct thermal sensitivities—e.g. photosynthesis, respiration and different nutrient cycles do not change at the same rate per degree of warming [57,60], yet individual processes can be relatively consistent over multiple scales and organizational levels. This is the case for respiration, for instance, which hints at a highly conserved and therefore predictable mechanistic basis [61–63]. Recent laboratory experiments across steep thermal gradients using riverine invertebrates have also shown that decomposition rates are determined primarily by the metabolic capacity of the assemblage, rather than species richness [22,64,65]. In subsequent experiments, greater levels of biodiversity were required to preserve overall functioning of multiple processes across a thermal gradient. However, performance curves differed among species and processes [65], implying that scope for insurance against climate change may be less than previously assumed.

**Figure 3.**
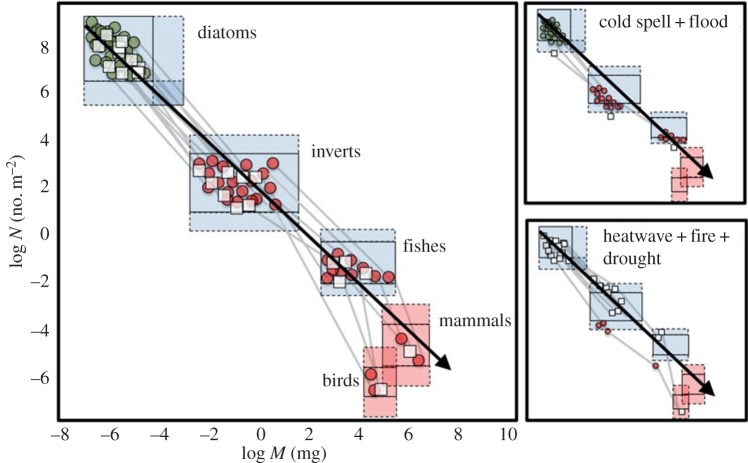
Conceptual diagram of shifts in body mass and abundance under two scenarios of combined extreme events (cf. figure 1). The white squares represent taxa associated with more lentic conditions, the coloured circles represent taxa associated with more lotic conditions. Green nodes represent producers, while red nodes represent consumers. The red boxes represent endotherms; the blue boxes ectotherms. Note, responses can be multifaceted and include species loss (especially among the higher trophic levels and endotherms), population declines (or occasionally increases, if predator release occurs), as well as changes in links and higher-level properties related to system complexity and energy flux.

**Figure 4.**
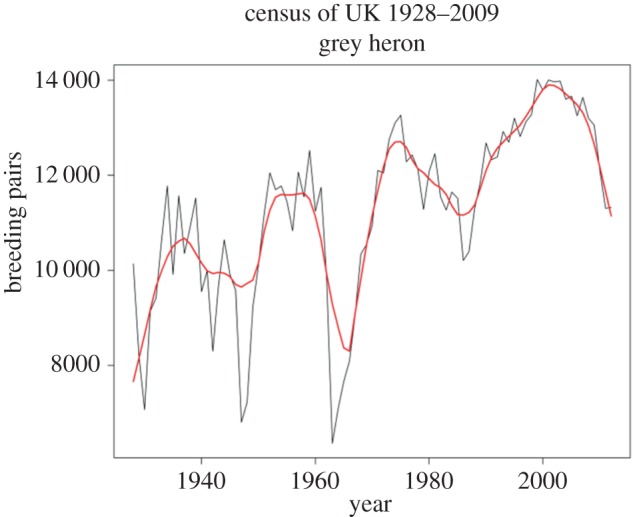
Fluctuations in abundance of an apex predator in a riverine ecosystem in response to environmental fluctuations and extreme events. The black line shows counts of grey heron breeding pairs in the UK 1928–2012. Red line is a LOWESS smoother. Note the particularly sharp drop following the exceptionally severe winter in 1963, which led to extensive and protracted freezing of inland waters (source: BTO).

Although warming is the dominant projection for future climate change, extreme cold spells are often predicted to increase in certain regions within this global trend (e.g. [66,67]). Unless they can hibernate, or move away, endotherms, which are already relatively scarce in fresh waters, will suffer disproportionately due to their need to maintain a higher mass-specific metabolic rate (figure 3). This is especially pronounced for riverine birds and bats, which have very high metabolic demands for their size (e.g. [65]), but even ectotherms, such as salmonid fishes, can suffer high mortality during cold spells [13]. Future modelling efforts therefore need to consider the effects of low-temperature extreme events as well. As many fish-eating birds and mammals also tend to be apex predators, extreme cold events will once again have skewed impacts within food webs, especially as the high metabolic costs of thermoregulation will be exacerbated when surface waters freeze, preventing access to prey. For example, the extremely cold winter of 1963 in the UK led to exceptionally high mortality among riverine birds, with widespread crashes in grey heron populations at the top of the food web due to starvation (figure 4): nearly half the national population was lost and it took many years to recover [68,69].

### (b) Hydrological fluctuations and extremes

Floods and droughts alter the distribution of water in the landscape through both time and space, and by extension how organisms interact with the environment and each other.

**Figure 5.**
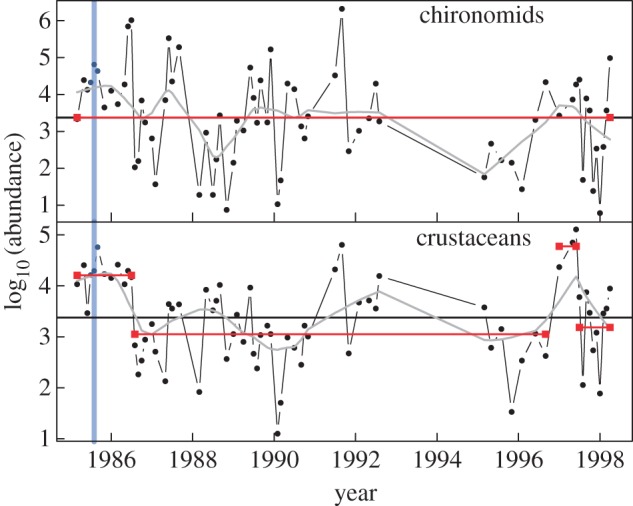
The correlated abundance of two trophic levels over 13 years following an extreme event in the Glenfinish River in Ireland. Abundance (number m^−2^) is in log_10_ scale. Significant break points in time series trend determined through circular binary segmentation analysis are shown by the red horizontal lines; the mean of all time series is shown by the black horizontal line. The grey line corresponds to a LOWESS smoothing. The vertical blue line indicates the catastrophic flood event in 1986 (adapted from [29]).

In general, droughts have much stronger ecological impacts than floods, which is perhaps unsurprising given that a surfeit of water seems less likely to be a problem than is a deficit for aquatic organisms [70–72]. A few riverine taxa, however, thrive under drought conditions, at least in the initial phases, as interactions with larger (predatory) species are weakened [14]. Some of the more *r*-selected taxa, such as certain chironomid species (figure 5), benefit from drought relative to the larger taxa higher in the food web [14,73]. This highlights that high dispersal ability, short generation times and large (meta-) population sizes, which are all linked to body size, can confer resilience [14]. Although our model does not capture all these traits or points of impact on food webs, because smaller organisms have higher recovery rates (high *r*_max_) from perturbation-induced rarity due to their mass-specific metabolic rates, the frequently observed pattern of selection of *r*-selected species is also seen in our model food webs, especially in nutrient-poor conditions (figure 2; electronic supplementary material, table S1 and figure S3).

**Figure 6.**
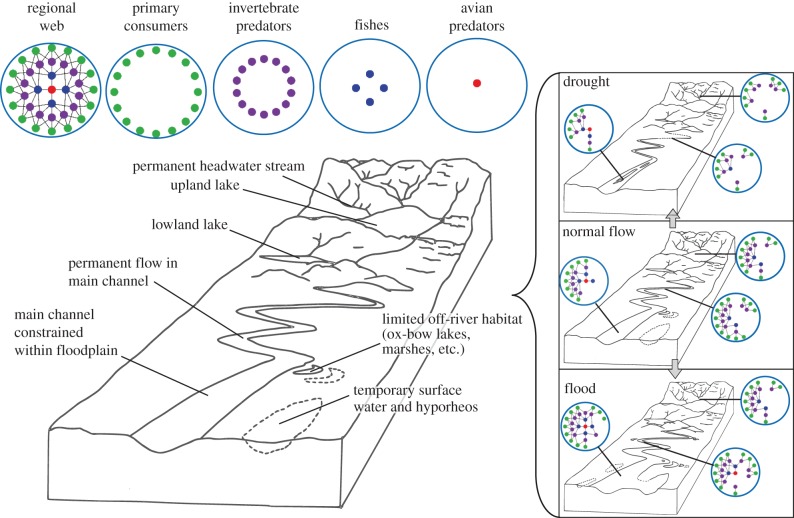
Hypothetical effects of extreme events on food web structure in a riverine landscape. The global web (top left) is altered by the hydrology of the system such that different regional (e.g. lower reaches and floodplain habitats) or local (e.g. individual headwater streams) communities are found under drought (top right), baseflow (middle right) or flood (bottom right) conditions. Note that increased habitat volume and beta diversity may increase regional web size under flood conditions, even if local webs may lose species.

Availability of physical refuges for organisms is also linked to body size, and we might expect a bimodal relationship between size and vulnerability to flood events, as small organisms can access interstitial refugia [74], whereas large powerful swimmers, such as adult salmonids may be able to withstand the flood or, as in the case of avian predators, avoid it by leaving the system entirely (figure 6). Intermediate-size organisms might suffer disproportionately, however, if they are too large to exploit small refugia or if they lack the physical or behavioural attributes or metabolic reserves to withstand the high flows during the flood's peak, and they are physically locked into the system (e.g. *Gammarus* shrimps versus chironomid midges; figure 5). Thus, larval fishes and the larger macroinvertebrates may be especially vulnerable, particularly those normally associated with more lentic habitats (e.g. many coarse fishes) in the lower reaches (figure 6). Again, some of these body size-driven effects are captured in our theoretical results (figure 2; electronic supplementary material, table S1 and appendix S1), but future work needs to explicitly account mechanistically for the effects of hydrological fluctuations on species interactions (figure 1).

At its most intense, drought leads to habitat loss and periodic drying of sediments, and field experiments have shown how effects can ripple through the food web [14,75,76], with density-dependent responses reflecting changes in the availability of wet refugia [30]. At larger scales, as the Murray–Darling River Basin (MDB) dried and became increasingly fragmented during the Big Dry, the ‘stream’ fauna became dominated by species with good dispersal abilities that were more typical of standing waters [77,78]. The effects of hydrological extremes and subsequent recovery are thus dependent upon the connectedness of suitable habitats and the food webs or ‘meta-networks’ they contain [47,79,80]. This spatial–temporal variation in food web structure is illustrated conceptually in figure 6.

Riverine communities are generally relatively resilient to flood events [17,81,82] as their invertebrate assemblages are typically dominated by highly mobile insects that can recolonize quickly. Many taxa have life history and/or behavioural responses cued to respond to particular precipitation patterns, including floods and high rainfall [83–87]. In addition, absolute flow size, timing of extreme events and flashiness are also key hydrological drivers of biotic impacts [88], probably exacerbated by habitat fragmentation [17,89].

Increases in the magnitude and flashiness of the largest floods, the areal extent of flooding and the frequency of high-flow events are widely predicted by many climate models [90,91]. This pattern appears to be supported by recent data [2,17], but relatively few small-scale experiments (less than 3 m^2^) have been conducted on the biotic effects of disturbance intensity, areal extent and their interaction [92–94], and large-scale replicated field experiments are still lacking [95–97]. There is evidence, however, that losses of in-stream flow refugia for the more lentic taxa may skew recovery rates [98–100]. A 1-in-100 year flood event in Alaska triggered severe declines in salmonids after overwintering eggs were washed out, although recovery was rapid [13]; whereas George *et al*. [28] showed a similarly extreme flood outside the spawning period during summer had no such effects. A rare example of long-term empirical data that captured a catastrophic summer flood in an Irish river [29,101] showed that most taxa took 4–10 years to recover, with the larger and less mobile species being the least resilient, in contrast to the small, abundant and aerial-dispersing chironomid midges, which were largely unaffected (figure 5).

### (c) Compound thermal and hydrological fluctuations and extreme events

Both droughts and floods can occur within the same system, and this may be repeated over a protracted period, as in the case of the MDB, whose naturally variable flow regime has become increasingly characterized by frequent droughts and floods. The decade-long ‘Big Dry’ (1997–2010) affected the entire basin [102]: its impacts included declines in fish and bird species at the higher trophic levels, as well as indirect changes in the food web due to release from predation [103,104]. The drought was finally broken by extensive flooding, which initially caused widespread fish mortality [105,106], although the return of a more normal flooding regime eventually led to increased zooplankton richness [107] and elevated fish spawning relative to the previous decade of drought [108].

Heatwaves, droughts and lightning storms can, when combined, trigger wildfires. These are extreme disturbances in their own right and are widely predicted to increase under future climate change [15,109–111] as temperatures rise [112]. The direct effects of wildfires on riverine ecosystems include increased water temperature and inputs of nutrients, charcoal and ash [18,113], with smaller headwaters being particularly susceptible. Biotic responses are often muted until the first flushing flows arrive (e.g. [111,112]), when indirect food web effects can become especially important [114,115]. Significant reductions in macroinvertebrate richness and density ([111,116; but see [113]) and the local extinction of fish populations are common responses [117]. Trophic generality typically increases following fires [118], even if the total numbers of species and links in the food web declines. Our theoretical predictions provide some insights into this—dynamically assembled food webs with low-nutrient inputs can respond to fluctuations very differently than those with nutrient subsidies (figure 2; electronic supplementary material, table S1 and appendix S1).

Invertebrates or amphibians may exhibit top-down release to become the dominant predators in the absence of fishes [119]. Burning can thus change the trophic basis of the food web, with detritus increasing in importance relative to algae [19,120]. Many of these shifts should be evident in transient responses in mass-abundance scaling within the food web, as larger taxa are lost and the effects of subsidies are reduced, with the scaling exponent steepening.

Consumers that are active dispersers are typically also fast recolonizers, whereas slow-dispersers, such as herbivorous snails, may take several years to recover despite being previously abundant [121]. Despite the protracted absence of certain nodes in the food web, most of the major trophic groups may return even within a matter of weeks, with the more resilient, small *r*-selected taxa (e.g. Chironomidae) often becoming dominant in this phase [115,122]. Our theoretical results are consistent with these patterns (figure 2; electronic supplementary material, table S1 and appendix S1), though we also find an interesting non-monotonicity (e.g. decrease and then increase in mean *r*_max_) in the response of population growth rates to environmental fluctuations, particularly at high carrying capacities. This suggests that nutrient subsidies can reverse or balance the trend towards *r*-selected organisms in dynamically assembling running-water food webs. These taxa are often dietary generalists that exert relatively weak top-down control, so algal blooms could potentially be triggered indirectly via the food web due to reduced grazing pressure in the absence of larger specialist herbivores. Essentially, as with drought and heatwaves, we should expect to see a general shift towards more bottom-up food webs dominated by short food chains and small species, as opposed to top-down, driven food webs (figure 2).

## 4. Discussion

Arguably, a mechanistic, metabolic approach is necessary for constructing a general framework to predict the effects of climatically driven environmental fluctuations in running-water ecosystems. Because, the system's responses at lower (individuals, interactions) levels of organization are easier to anticipate from ‘first’, mechanistic principles, we have presented and illustrated a framework that takes this relative predictability and scales it up to the dynamics of the whole ecosystem through a series of steps. By doing so, we may be better able to understand why ecosystem-level properties seem to be more resilient (and resistant) than those of local community food webs, not just in running waters, but in aquatic ecosystems in general. This has often been ascribed to the redundancy of species or individuals within interaction networks [29,123,124]. However, our predictive capacity at the community level is particularly limited when we seek to predict *which* species are affected, leading many ecologists to abandon the Latin binomial, and to focus on other community-level properties that are predictable (e.g. species–area relationships, mass-abundance allometries) and based on general ecological theory, while avoiding many of the complicating effects of taxonomic and biogeographic differences among systems (figures 1, 3 and 6).

Within riverine food webs, energy typically flows along a series of food chains from small, abundant invertebrate primary consumers to larger, rarer vertebrate predators (e.g. [14,125,126]) and such mass-abundance scaling can be explored using size-spectra, individual-size distributions and trivariate food web approaches. Deviations from the normal (or predicted) state should become larger as an event becomes more extreme and the typical scaling rules are distorted by external transient stressors (by changes in size distributions; figures 2 and 3). Such approaches have been used recently to gauge the impacts of various perturbations in freshwaters, including both warming [57,127] and drought [76]. In the latter case, as predicted, drought altered stream food webs primarily via the loss of large and/or rare species and especially those that were rare for their size—i.e. those below the community-wide mass-abundance regression line may have already been in suboptimal niche space prior to their extirpation from the food web by drought [76].

These approaches can help to connect higher-level structural and functional responses of relevance to extreme events, such as changes in network complexity (e.g. species and/or link richness) and whole-system metabolism [60,127,128]. At the ecosystem level, much of the taxonomic complexity at the community level becomes extraneous as an explanatory variable, and simpler rules may apply. Consistent responses in ER, for instance, to temperature change [60,62,129], despite huge levels of taxonomic turnover, are suggestive of prevalent functional redundancy. This implies that at least some ecosystem processes might be underpinned by relatively simple physiological and metabolic constraints [56], which offers cause for optimism for predicting responses to extreme events.

We emphasize that the baseline physical and biotic variability of these highly dynamic systems needs to be quantified to gauge their stability. However, this can vary across organizational levels: communities or food webs may be resilient, even if some of their constituent populations or pairwise interactions are not. A considerable body of theory has developed around the idea that running waters are inherently dynamic systems (e.g. the flood pulse concept [130], patch dynamics and flow refugia [131], etc.) yet this aspect of disturbance ecology has developed unconnected to any explicit consideration of the role of metabolic constraints, species interactions and food web dynamics, which we now know are also important filters that mould riverine ecosystems and their responses to stressors (e.g. [119,121]). The impacts of fluctuations on running waters can therefore be better viewed by combining disturbance ecology, which provides an important conceptual and empirical context, and food web ecology, which recognizes the complex and multiple dimensions of biodiversity. Many of the aspects of disturbance ecology as applied to running waters are conceptual and phenomenological models, rooted to varying degrees in empirical data, whereas more formal mathematical approaches based on first principles remain scarce. Both approaches have merit, but by blending them we argue important new insights are likely to emerge, as well as greater predictive power for dealing with future events that are increasingly likely to be outside what previous data have captured.

### (a) Future empirical and theoretical directions

Although many organismal traits (e.g. high dispersal ability, short generation time) and higher level attributes (e.g. large population size) that confer resilience to extreme events are related to body size, others might not be obviously size-dependent. The relative importance of these different traits could, in theory, be measured as the deviation from a general size–metabolism relationship (e.g. figure 3), which could then be used to assemble indices of sensitivity to extreme events. Traits that are independent of body size (e.g. biochemical adaptations that prevent body fluids from freezing) should have the largest residuals relative to the general mass–metabolism relationship, so such approaches could help to identify the number of dimensions involved in trait space as well as important exceptions to the general rules, to simplify the numbers of drivers and responses that need to be considered [76]. In addition, we also need better and more extensive data on the thermal physiology of riverine organisms. Along with data and models for the effect of size on individual physiology and performance, data and models for thermal performance curves are necessary for accurately scaling up metabolic constraints, and for accounting for factors particularly relevant to riverine systems, such as patterns of physiological mismatches [27] (figure 1).

Another aspect that has been largely overlooked is the altered dimensionality of the habitat that will occur under extreme events relative to baseline conditions, as the system becomes essentially more 2D (droughts) versus 3D (floods), and this will alter the strength of species interactions ([43]; figure 1). In addition, ice formation can reorganize the physical habitat of the river bed during extreme cold spells [132,133], and the wider ramifications of this potential interplay between physical habitat change and the concomitant metabolic costs remains unknown. We also need to be able to look beyond the river's banks and to address how extreme events might alter terrestrial–aquatic linkages in the landscape—for instance, following a wildfire the first post-fire storm often triggers sediment erosion and mobilization, which can lead to the loss of catchment vegetation [113,134]. This can also change stream thermal regimes, particularly in forested systems, so there may be metabolic consequences that persist long after the fire has passed [135,136]. When a wildfire removes the riparian vegetation, the food web undergoes dramatic restructuring: invertebrate shredders decrease and collector-gatherers, predators and scrapers increase following the loss of predatory fishes [137]. Fish (re)colonization is commonly constrained by dispersal barriers rather than food resources, and many are very sensitive to fire [136], but for those that survive, their trophic interactions will shift depending on whether riparian vegetation remains intact or not. If it is completely burnt, aquatic invertebrates become more prevalent prey items as terrestrial invertebrates decline [138,139]. This could intensify in-stream predation temporarily, but at longer time scales top-down pressure should ultimately fall if terrestrial subsidies cannot maintain fish populations at their previous (elevated) levels [140].

Metabolic approaches could therefore also be useful in dealing with impact on the relative importance of terrestrial subsidies of food and/or habitat, as these will elevate the recipient population densities such that they will sit above the general mass–abundance scaling relationship. As these links become impaired, so the affected species should become more tightly coupled to the general relationship that describes the solely aquatic system. New generations of models based on first principles will also be needed if we are to anticipate and respond to future scenarios, especially as many of those phenomena and the novel communities they will produce have not yet been seen: today's extremes may very well be tomorrow's means, as our baselines continue to shift ever further from what we have known in the past. Extreme events are difficult and expensive to mimic in large-scale field experiments, so empirical multi-species studies are still scarce and small in scale [129,141], yet models and mesocosm studies could help bridge the data–theory gap in the interim until more realistic larger-scale manipulations are feasible [142].

### (b) Ecosystem services, synergies and interactions with other anthropogenic impacts: the real-world context of environmental fluctuations

As we now move from our more mechanistic considerations into the realm of ecosystem services, our current lack of knowledge becomes ever-more apparent, although we can still generate some plausible hypotheses for future testing. This is important because extreme events can have devastating socioeconomic costs. For instance, the MDB is Australia's largest river system, it produces over 70% of the country's irrigated crops, supports over 2 million people and contains internationally significant freshwater ecosystems [143], and the Big Dry has been estimated to have cost more than 810 million USD [144]. As the threat of climate events grows, social systems will aim to alter catchment properties to mitigate the impacts (e.g. larger storm events tend to increase use of drains, whereas droughts increase the adoption of irrigation). Consequently, responses to climate events ultimately lead to changes in land use practices that can amplify (or dampen) the effects on water flows and quality in riverine landscapes. These climate-related stressors also need to be set in the wider context of how they will interact with the many other existing and emerging stressors in the world's running waters [145–147], whose combinations are unlikely to act additively [148]. The extensive modification of river channels for drainage, irrigation, flood protection and urban development means that pristine waterbodies are increasingly hard to find and extreme events are likely to further amplify the total amount of stress imposed on a given system [149–152].

More severe floods are likely to trigger further manipulations of river geomorphology to protect humans and infrastructure, which could accelerate reductions in habitat heterogeneity and quality [17,153]. Whether the communities in these highly modified habitats respond to extreme events in ways comparable to more pristine communities remains a moot point [17,96], and the need to develop approaches based on first principles, rather than (unknown) phenomenological conditions, is once again highlighted.

Land use and human actions at the larger, catchment scale filters local in-stream biota and its responses to extreme events. The use of freshwaters for drinking water, industry and irrigation has already altered the hydrology of most of the world's river systems [146], and overabstraction is a growing problem that often exacerbates the effects of extreme events—e.g. human use for crops and industry during protracted summer droughts can lower the water table by several metres. Chemical stressors may also be influenced by extreme events, as temperature affects thermodynamics, and fluxes or concentrations of solutes are determined hydrologically; storm events exacerbate acid pulses in base-poor upland streams [154], which can alter the food web and ecosystem processes [155,156] and may even be hindering biological recovery in the face of several decades of reduced acidifying emissions [157].

Centuries of anthropogenic impacts have almost certainly compromised the ability of riverine communities to absorb the additional impacts of extreme events [158–160], and freshwater ecologists are focusing on how to manage ecosystem processes and services more sustainably in the future. Because ecosystem resilience is determined by both the sensitivity of the system to disturbance and its buffering capacity, identifying stabilizing mechanisms will improve future risk management strategies for coping with extreme events.

Catchment management has mostly been geared towards delivering services with high market value, such as food and fibre [161], although the floodplain's role for absorbing floodwaters and how in-stream habitat heterogeneity can improve fish production are now being recognized [162]. These changing perceptions might help to reintroduce some of the lost resilience of these systems [17]: restoring riparian vegetation could create thermal refugia [163,164] to help offset the rising threat of future heatwaves. Such restorative management has potential for protecting multiple components of the food web and associated ecosystem processes [81,165,166]. In many regions, environmental water reserves are already being allocated to provide sustaining flows [167] that could help buffer extreme events by providing refugia in the coming decades [168,169]. Climate change and extreme events will probably have dramatic effects on the extent and connectivity of freshwater habitats (figure 6), which could be managed at landscape scales through the provision of environmental flows or through protection of existing, or construction of new, refugial habitats. The next step is to be able to identify suitable systems and scales in time and space [170].

In conclusion, it is clear that environmental fluctuations and extreme events have long been overlooked in the context of climate change, relative to other stressors, in running waters. The gaps in our understanding are still multifaceted and serious, especially as most of the world's population lives on floodplains. However, even though sustained long-term and large-scale monitoring is still the exception rather than the rule, much progress can still be made by combining space-for-time substitutions, modelling, and experimental approaches to push the field forward. Indeed, we are now at an unprecedented juncture in the broader field of ecology, where theoretical and empirical advances are making it possible to develop a mechanistic yet general model of ecosystem dynamics and confront these with empirical data. Running waters, owing to their dynamic nature, offer a substantially more complex empirical and theoretical challenge in this regard. We hope this paper and the conceptual framework that we have developed stimulates better-coordinated and focused theoretical and empirical efforts to tackle this challenge.

## Supplementary Material

Supplementary material
