# Supplementary material for: The effects of climatic fluctuations and extreme events on running water ecosystems
Source: Philos Trans R Soc Lond B Biol Sci. 2016 May 19;371(1694):20150274. doi: 10.1098/rstb.2015.0274 (PMC4843695; doi:10.1098/rstb.2015.0274)
Supplement: Supplementary material [file rstb20150274supp1.pdf]

Woodward G *et al.* 2016 The effects of climatic fluctuations and extreme events on running water ecosystems. *Phil. Trans. R. Soc. B* **371**: 20150274.
http://dx.doi.org/10.1098/rstb.2015.0274

Supplementary Material

Appendix 1: Model structure and parameterizations

We use a discrete-time specification of main text eqn 2.3 in order to include the effects of environmental stochasticity and extreme events. Consider a food web of S interacting species' populations. Each species' population x is assumed to grow according to the discrete-time, generalized Lotka-Volterra specification of main text eqn 2.3. In this specification, the ith species' population is governed by the equation:

$$x_{i,t+1} = x_{i,t} + r_i x_{i,t}\left(1 + \frac{\sum_{j=1}^{n}(a_{ij}x_{j,t})}{K_i}\right) \quad \text{(S1)}$$

where x_i is biomass density of the ith species (node value), r_i its per-capita intrinsic or maximal (r_{max}) growth rate and K_i its carrying capacity, which determines the strength of intra-specific density dependence and equilibrium population sizes in the absence of interspecific interactions. For the jth consumer and i^{th} resource, coefficient a_{ij} is mass-specific search rate which governs the rate of per-unit biomass loss of the ith species to consumption by the jth species [1]. Finally, the coefficient a_{ii} (when $i = j$ in the sum in eqn (S1)) is a mass-specific intraspecific "search" rate that governs intraspecific interference between individuals of the ith species [1,2].

Thus biomass gain rate of the former (a_{ji}) and loss of the latter (a_{ij}) are related such that,

$$a_{ji} = -ea_{ij}, \text{ (when } j \text{ consumes } i\text{)} \quad \text{(S2)}$$

where e is the consumer's biomass conversion efficiency, which does not scale with body mass within major organismal groups or trophic levels [3,4]. Henceforth we will assume $e = 0.5$, close to the value observed for carnivores. As long as $e > 0$, this paper's results do not qualitatively depend upon it. We model fluctuations in total population size as follows:

$$x_{i,t+1} = x_{i,t}\left[1 + r_i\left(1 + \frac{\sum_{j=1}^{n}(a_{ij}x_{j,t})}{K_i}\right)\right](1 + \varepsilon_{i,t}) \quad \text{(S3)}$$

where ε_t is a random variable with mean = 0 and variance σ_ε^2 [5,6]. This introduces density-dependent environmental stochasticity in population sizes — larger populations experience a proportionally larger change in density. This would be the case if the fluctuation affected a fundamental rate such as r_{max}, such as through a temperature-driven impact on physiology [7,8]. The model can easily be extended to directly include fluctuations in temperature-dependent life history [8,9] or interaction [10,11] parameters. These fluctuations may be correlated between populations if populations tend to have a similar thermal sensitivity, for example, which we model using a multivariate Gaussian process having a characteristic correlation between each population's perturbations, but uncorrelated with respect to time (no temporal auto-correlation). In other words, all populations may experience "good" and "bad" in a perfectly correlated manner (correlation = 1), or completely independently (correation = 0), but for any two time steps t and k, ε_t is independent of ε_k. A low correlation implies a poor high physiological mismatch between species.

© The Authors under the terms of the Creative Commons Attribution License http://creativecommons.org/licenses/by/3.0/, which permits unrestricted use, provided the original author and source are credited.

***Extreme events*:** Populations may also experience catastrophic declines due to sudden changes in conditions (extreme events), such as the advent of droughts or temperature spikes. The impact of such events can be both density-independent as well as dependent. For example, in the case of temperature change, metabolic stress would lead to density-independent mortality, whereas concurrent reduction in trophic resources would increase competition in larger populations, resulting in density dependent mortality. For simplicity, here we model only the density-dependent scenario by adding a probabilistic catastrophe term as an instantaneous reduction by a fraction δ in all populations (Lande 1993):

$$x_{i,t+1} = \begin{cases} f(x_{i,t})(1-\delta) \text{ with probability } p_\delta, \\ f(x_{i,t}) \text{ otherwise} \end{cases} \quad \text{(S4)}$$

Where $f(x_{i,t})$ is given by the right hand size of eqn (S3), and p_δ is the per-time step probability of an extreme event. This allows us to model extreme events occurring at different characteristic frequencies. We now include metabolic constraints into the food web model (eqn (S3)) as follows. For simplicity, we will focus only on the size (and not temperature) components of these metabolic constraints, leaving the stochastic fluctuation and extreme events parameters in eqns (S3) & (S4) to simulate, albeit indirectly, the effect of thermal fluctuations.

Size-scaling parameterizations

For intrinsic growth rate we use

$$r_i = r_0 m_i^{\beta-1} \quad \text{(S5)}$$

where r_0 is a normalization constant that includes the effect of temperature (main text eqn 2.1), m is the species' average adult body mass, and $\beta = 0.75$. These relationships are empirically well supported [8,9,12,13], and have been used previously in similar contexts [1,14–17]. For the search rate coefficient between the ith resource and j^{th} consumer species we use

$$a_{ij} = -a_0 m_j^{-0.25} \varphi_{ij} \quad \text{(S6)}$$

where, a_0 is a normalization constant, and $\varphi_{ij} \in [0, 1]$ is a dimensionless function that embodies attack success probability. Thus from eqn (S2),

$$a_{ji} = e a_0 m_j^{-0.25} \varphi_{ij} \quad \text{(S7)}$$

Note that in eqns (S6)–(S7), the commonly used quarter-power exponent that has been used for the scaling of search rate, as in numerous previous studies [15–18]. According to the results of Pawar et al [1,11,19,20], this is approximately the scaling exponent for 2*D* (two spatial dimensions; e.g., benthic) interactions only. For simplicity, we am thus assuming here that all food webs have 2*D* interactions only [e.g., 21]. Future work should consider the effect of 3*D* or a mixture of 2*D*-3*D* interactions (main text Fig. 1). Next, we assume that attack success probability is unimodal ([22]) with respect to average resource mass (consumer-resource body mass ratio, or size-ratio):

$$\varphi_{ij} = \exp\left(-\left(s\log\left(m_j m_i^{-1}/k\right)\right)^2\right) \quad \text{(S8)}$$

Here s determines how rapidly the function reaches its peak k, the size-ratio of maximum consumption rate. This is a simplification of the potentially complex dynamics of consumer-resource encounter and consumption rates in nature, but captures an important feature of empirically observed attack and capture rates — these are unimodal because attack success decreases and handling time increases at extreme size-ratios [23–25]. The actual values of k and s are expected to vary with type of consumption (foraging) strategy as well as habitat type. For example, in the case of predator-prey interactions, because smaller organisms have greater mass-specific power relative to larger ones, and can hence handle a larger range of prey sizes, k may be closer 1 (or even <1) for small consumers, and s smaller (a more gradual decline of consumption rate at extreme ratios). Brose et al. [26] have shown that invertebrate consumers do indeed have a k closer to 1 than vertebrates across disparate habitat types, suggesting their superior ability to attack and capture prey closer to their own size. Our results are qualitatively insensitive to a wide range of choice of parameters k and s (Table S2).

Stochastic community assembly under environmental fluctuations

We simulate open, dynamically assembling food webs till the system reaches immigration-extinction equilibrium (IEE) [22,27] as follows:

- *Immigration*. Beginning with the establishment at least one basal species, with some per-time-step probability p_c, a species' population is introduced at an extinction threshold biomass abundance x_e. Each immigrant species is generated by sampling a body size from a Beta(1,ω) distribution that captures the qualitative properties of empirical body size distributions [22]. Inter- and intra-specific interaction parameters are determined by body sizes (eqns (S5)–(S8)).
- *Trophic linking*. Upon colonization, the jth immigrant establishes a trophic link to the ith pre-existing one with a connectance probability p_{ij}. For each assembly simulation, conditional upon p_{ij}, a "vulnerability probability" p_v ranging between 0.5–1 is set: $p_v = 0.5$ means that the jth immigrant is equally likely to be a resource or a consumer of the ith resident species (provided it was not basal), while $p_v = 1$ meant that the jth immigrant can only be a consumer.
- *Interaction driven extinction*. At every time step, a species is deleted from the system if its density drops below x_e.

This simulation is continued till the system reaches IEE. Simulations were performed in Python with scipy. Simulation parameters were chosen as follows [22,28] (Table S2):

- e was fixed at 0.5 for all species, the approximate midpoint of the range reported from empirical data [9,13]. Values ranging from 0.1–1 don't change the simulation results qualitatively.
- p_v was set to 0.9 because this is the midpoint of the range [0.75–1] that yields communities with structural and dynamical characteristics similar to that of real ones, similar to the niche model [28]. The results do not change qualitatively over the full range of [0.5–1].
- x_e was set to 10^{-20}; the results do not change qualitative for values ranging from $10^{-32} – 10^{-3}$.

In addition, body size related simulation parameters were chosen as follows:

- ω, which determines the shape of the immigrant pool size distribution was varied between 1 (uniform distribution; immigration rate independent of body size) and 2 (power law-like with slope = –2; immigration probability decreases with body mass). This range of ω was chosen because: (i) Empirical data show the distributions of sizes at large spatial scales are right-skewed [29,30], probably partly driven by speciation rate, which appears to follow a negative power law relationship with body size [31–33], possibly linked to metabolic scaling [34–36] and, (ii) dispersal ability should increase with body size [13,37]. Only considering (i) means that ω should be > 1; choosing $\omega = 2$ sets a reasonably high upper limit to this bias. Considering (ii) means that the effect of (i) may be somewhat negated due to dispersal ability. However, because speciation within the local community also adds to the effective bias towards immigration by smaller species and because data on dispersal ability itself is biased towards larger organisms [13], it is unlikely that (ii) can overwhelm the effects

of (i). Hence $\omega = 1$, which yields the uniform distribution expected if the effects of (i) and (ii) exactly cancel out, is a reasonable lower limit to the immigration rate bias [22].

- The log-body mass range $[y'_{\min}, y'_{\max}]$ was chosen to be [–12,12] because this is approximately the range of species' log-body masses observed across empirical communities [26] (also see [22]).
- k was chosen to vary randomly between 10^{-3} and 10^{3} with uniform probability (consumers 1000 times smaller to 1000 times larger than resources), which covers most of the range considered to be "optimal" (in the sense of viable, or evolutionarily stable strategy for the consumer) in previous studies on consumer-resource interactions [14,38], and accommodates potential differences in k across the most common trophic interaction types seen in food webs (i.e., predator-prey, herbivore-plant and parasitoid-host) [26].
- The parameter s was set to 0.1; however, varying it between a wide range (0.05–0.5) does not change the results (results not shown; also see [22]).
- The allometric constant r_0 was chosen to be 1.
- The search-rate scaling constant a_0 was varied between 10^{-3}–1 based upon recent empirical results [1] — the results shown here are for $a_0 = 1$. Other values in this range do not change the results qualitatively.
- a_{ii} was chosen according to the target mean n at IEE; larger values give larger feasible communities [6,21,22,39].

Food web properties. At IEE, we calculated various metrics, including stability properties (Main text Fig. 2 & Table S1; Fig S1-4). For stability properties, we calculated the Jacobian matrix J, which is the S x S matrix of partial derivatives,

$$J = \begin{pmatrix} \frac{\partial f_1(\bullet)}{\partial x_1} & \cdots & \frac{\partial f_1(\bullet)}{\partial x_S} \\ \vdots & \ddots & \vdots \\ \frac{\partial f_S(\bullet)}{\partial x_1} & \cdots & \frac{\partial f_S(\bullet)}{\partial x_S} \end{pmatrix}$$

The local stability criterion of the system is that the real parts of all the S eigenvalues, $\lambda_{i=1,\dots,S}$ of $\mathbf{C}$ lie in the left half of the plane of complex numbers, i.e., $max\ [\lambda_{i=1,\dots,S}] = \lambda_{max} < 0$. We also calculated the system's "dynamic dimensionality", D — the number of eigenvalues required to account for 95% of the total sum of the eigenvalues. This is a measure of the number of dimensions that determine the system's return time and hence is indicative of the amount of dynamical redundancy, which we define as $R = D^{-1}$, i.e., greater the dimensionality, the lower its dynamical redundancy.

Food web characteristics: We now identify and describe a set of topological and non-topological features of networks that were used to characterize emergent and evolving food webs.

Distribution of $r_{\max}$*'s and body sizes*: The $r_{\max}$ of each population was calculated given its size using eqn (S5). We calculated two summary statistics about both size and growth rate distributions in the emergent food webs: the mean and the skewness.

Degree and degree distributions: We measured the degree of the directed, weighted food-webs by converting the corresponding adjacency matrix A to an unweighted, binary (but directed) one A^{UB} with its $n(n-1)$ off-diagonal elements given by,

$$a_{ij}^{UB} = \begin{cases} 1 \text{ if } |a_{ij}| > 0, \\ 0 \text{ otherwise} \end{cases}$$
$$a_{ji}^{UB} = \begin{cases} 1 \text{ if } |a_{ji}| > 0, \\ 0 \text{ otherwise} \end{cases} \quad \text{(S9)}$$

The degree of each node was then calculated from A^{UB} as a sum of its in- and out-degree. From this, the average degree (k_{av}), was calculated.

Connectance: We measure connectance as,

$$C = \frac{\sum_{i,j=1}^{n} a_{ij}^{UB} + a_{ji}^{UB}}{n^2} \quad \text{(S10)}$$

where a_{ij}^{UB} and a_{ji}^{UB} are directed edges between the ith and the jth nodes.

Trophic levels: Using the unweighted version of the interaction matrix, we calculated the shortest path length G_{ij} between each *i-j* vertex pair ([40]). The average shortest path length, G_{av} was then calculated as the average of all the G_{ij}'s. Here because A^{UB} represents a directed graph, we calculated G_{av} *as,*

$$G_{av} = \frac{\left(\sum_{i,j=1}^{n} G_{ij} + G_{ji}\right)}{n(n-1)} \quad \text{(S11)}$$

References

1. Pawar, S., Dell, A. I. A. & Savage, V. M. V. 2012 Dimensionality of consumer search space drives trophic interaction strengths. *Nature* **486**, 485–489.
2. DeLong, J. 2014 The body-size dependence of mutual interference. *Biol. Lett.* **10**.
3. Hechinger, R. F., Lafferty, K. D., Dobson, A. P., Brown, J. H. & Kuris, A. M. 2011 A common scaling rule for abundance, energetics, and production of parasitic and free-living species. *Science (80-.).* **333**, 445–8.
4. DeLong, J. P., Okie, J. G., Moses, M. E., Sibly, R. M. & Brown, J. H. 2010 Shifts in metabolic scaling, production, and efficiency across major evolutionary transitions of life. *Proc. Natl. Acad. Sci. U. S. A.* **107**, 12941–5.
5. Lande, R., Engen, S. & Sæther, B. E. 2003 *Stochastic population dynamics in ecology and conservation*. Oxford: Oxford University Press.
6. May, R. M. 1974 *Stability and Complexity in Model Ecosystems*. Princeton, NJ: Princeton University Press.
7. Dell, A. I., Pawar, S. & Savage, V. M. 2011 Systematic variation in the temperature dependence of physiological and ecological traits. *Proc. Natl. Acad. Sci. U. S. A.* **108**, 10591–10596.
8. Savage, V. M., Gilloly, J. F., Brown, J. H., Charnov, E. L., Gillooly, J. F. & West, G. B. 2004 Effects of body size and temperature on population growth. *Am. Nat.* **163**, 429–41.
9. Brown, J. H., Gillooly, J. F., Allen, A. P., Savage, V. M. & West, G. B. 2004 Toward a metabolic theory of ecology. *Ecology* **85**, 1771–1789.
10. Dell, A. I., Pawar, S. & Savage, V. M. 2014 Temperature dependence of trophic interactions are driven by asymmetry of species responses and foraging strategy. *J. Anim. Ecol.* **83**, 70–84.
11. Pawar, S., Dell, A. I. & Savage, V. M. 2015 From metabolic constraints on individuals to the dynamics of ecosystems. In *Aquatic Functional Biodiversity: An Ecological and Evolutionary Perspective* (eds A. Belgrano G. Woodward & U. Jacob), pp. 3–36. Elsevier.
12. McCoy, M. W. & Gillooly, J. F. 2008 Predicting natural mortality rates of plants and animals. *Ecol. Lett.* **11**, 710–716.
13. Peters, R. 1983 *The ecological implications of body size*. 1st edn. Cambridge: Cambridge University Press. [cited 2014 Mar. 10].
14. Weitz, J. S. & Levin, S. A. 2006 Size and scaling of predator-prey dynamics. *Ecol. Lett.* **9**, 548–57.
15. Yodzis, P. & Innes, S. 1992 Body size and consumer-resource dynamics. *Am. Nat.* **139**, 1151–1175.
16. Brose, U., Williams, R. J. & Martinez, N. D. 2006 Allometric scaling enhances stability in complex food webs. *Ecol. Lett.* **9**, 1228–1236.
17. Virgo, N., Law, R. & Emmerson, M. 2006 Sequentially assembled food webs and extremum principles in ecosystem ecology. *J. Anim. Ecol.* **75**, 377–86.
18. Otto, S. B., Rall, B. C. B. C. & Brose, U. 2007 Allometric degree distributions facilitate food-web stability. *Nature* **450**, 1226–1229.
19. Giacomini, H. C., Shuter, B. J., de Kerckhove, D. T. & Abrams, P. a. 2013 Does consumption rate scale superlinearly? *Nature* **493**, E1–E2.
20. Pawar, S., Dell, A. I. & Van M. Savage 2013 Pawar et al. reply. *Nature* **493**, E2–E3.
21. Tang, S., Pawar, S. & Allesina, S. 2014 Correlation between interaction strengths drives stability in large ecological networks. *Ecol. Lett.* **17**, 1094–1100.
22. Pawar, S. 2015 The Role of Body Size Variation in Community Assembly. *Adv. Ecol. Res.* **52**, 201–248.
23. Persson, L., Leonardsson, K., de Roos, A. M., Gyllenberg, M. & Christensen, B. 1998 Ontogenetic Scaling of Foraging Rates and the Dynamics of a Size-Structured Consumer-Resource Model. *Theor. Popul. Biol.* **54**, 270–293.
24. Brose, U., Ehnes, R. B., Rall, B. C., Vucic-Pestic, O., Berlow, E. L. & Scheu, S. 2008 Foraging

theory predicts predator-prey energy fluxes. *J. Anim. Ecol.* **77**, 1072–8.
25. Aljetlawi, A. A., Sparrevik, E. & Leonardsson, K. 2004 Prey-predator size-dependent functional response: derivation and rescaling to the real world. *J. Anim. Ecol.* **73**, 239–252.
26. Brose, U. et al. 2006 Consumer-resource body-size relationships in natural food webs. *Ecology* **87**, 2411–2417.
27. Pawar, S. 2009 Community assembly, stability and signatures of dynamical constraints on food web structure. *J. Theor. Biol.* **259**, 601–612.
28. Pawar, S. 2009 Community assembly, stability and signatures of dynamical constraints on food web structure. *J. Theor. Biol.* **259**, 601–612.
29. Allen, C. R., Garmestani, a S., Havlicek, T. D., Marquet, P. a, Peterson, G. D., Restrepo, C., Stow, C. a & Weeks, B. E. 2006 Patterns in body mass distributions: sifting among alternative hypotheses. *Ecol. Lett.* **9**, 630–43.
30. Clauset, A. & Erwin, D. H. 2008 The evolution and distribution of species body size. *Science (80-.).* **321**, 399–401.
31. Dial, K. P. & Marzluff, J. M. 1988 Are the smallest organisms the most diverse? *Ecology* **69**, 1620–1624.
32. Marzluff, J. M. & Dial, K. P. 1991 Life-history correlates of taxonomic diversity. *Ecology* **72**, 428–439.
33. Kozlowski, J. & Gawelczyk, A. T. 2002 Why are species' body size distributions usually skewed to the right? *Funct. Ecol.* **16**, 419–432.
34. Allen, A. P. & Gillooly, J. F. 2006 Assessing latitudinal gradients in speciation rates and biodiversity at the global scale. *Ecol. Lett.* **9**, 947–954.
35. Allen, A. P., Gillooly, J. F., Savage, V. M. & Brown, J. H. 2006 Kinetic effects of temperature on rates of genetic divergence and speciation. *Proc. Natl. Acad. Sci. U. S. A.* **103**, 9130–9135.
36. Allen, A. P. & Savage, V. M. 2007 Setting the absolute tempo of biodiversity dynamics. *Ecol. Lett.* **10**, 637–646.
37. Hein, A. M., Hou, C. & Gillooly, J. F. 2012 Energetic and biomechanical constraints on animal migration distance. *Ecol. Lett.* **15**, 104–110.
38. Cohen, J. E. 2008 Body sizes in food chains of animal predators and parasites. In *Body size: the structure and function of aquatic ecosystems* (eds A. G. Hildrew D. G. Raffaelli & R. Edmonds-Brown), pp. 306–325. Cambridge, UK.: Cambridge University Press.
39. Pawar, S. S. 2009 Community assembly, stability and food web structure.
40. Newman, M. E. J. 2003 The structure and function of complex networks. *Siam Rev.* **45**, 167–256.

Table S1: Summary of qualitative theoretical results of the food web assembly modelling, comparison with previous theoretical work, and empirical support (for or against). An asterisk (*) indicates that the previous theoretical or empirical study supports or partly supports our results. See main text for references, except for the following which do not appear there: Ledger, M. E., Harris, R. M., Armitage, P. D., & Milner, A. M. (2012). Climate Change Impacts on Community Resilience: Evidence from a Drought Disturbance Experiment. Advances in Ecological Research, 46, 211 : Ledger, ME, Milner, AM, Brown, LE, Edwards, FK, Hudson, LN and Woodward, G (2013b) Extreme Climatic Events Alter Aquatic Food Webs. A Synthesis of Evidence from a Mesocosm Drought Experiment. Advances in Ecological Research, 48. 343 – 395; McHugh, P. A., Thompson, R. M., Greig, H. S., Warburton, H. J., & McIntosh, A. R. (2015). Habitat size influences food web structure in drying streams. Ecography, 38, 700-712; Jellyman, P. G., McHugh, P. A., & McIntosh, A. R. (2014). Increases in disturbance and reductions in habitat size interact to suppress predator body size. *Global Change Biology*, *20*, 1550-1558.

Property	Effect of fluctuations	Theoretical support	Empirical evidence	
			Other aquatic systems	Running waters
Species richness	↓	(Bastolla et al. 2005, Lehmann-Ziebarth and Ives 2006)*	NA	Ledger et al 2011*, 2013a,b*; McHugh et al 2015*
Mean body mass	↓	NA	NA	Woodward et al 2012*; Jellyman et al 2015*
Mean consumer-resource body mass ratio	↑	NA	Jennings and Warr 2003*	Woodward et al 2012*
**Intrinsic growth rates**	↑	NA	NA	Lancaster & Ledger 2015*
**Connectance**	↓	Lehmann-Ziebarth and Ives 2006*	Briand 1983*	Ledger et al 2013a,b
**Trophic levels**	↓	Bastolla et al. 2005*	(Briand and Cohen 1987)	Woodward et al 2012*; McHugh et al 2015*
**Mean degree (generality)**	↓	NA	NA	Ledger et al 2013a,b
**Resilience**	↑	May 1973, Lehmann-Ziebarth and Ives 2006*	NA	Ledger et al 2012*; Woodward et al 2015*

Table S2. Model parameters for simulations of a stochastically assembling food web under environmental fluctuations. The results of the model's simulations remain qualitatively unchanged for the ranges of parameter values shown.

Parameter	Description	Value
S	Number of populations in the food-web	state variable
x_i	i^{th} species' population size	state variable
x_e	Population size (biomass density) at colonization (extinction occurs below this size)	$10^{-10} – 10^{-1}$
m_i	i^{th} species' body size	Sampled from gamma distribution
ω	Distribution parameter for body sizes in the global immigration pool	1 – 2
β	Scaling exponent for mass-metabolism allometry	0.75
r_0	Scaling constant for intrinsic rate of biomass production	1
K	Location parameter for the function φ_{ij}	0.001–1000
S	Scale parameter for the function φ_{ij}	0.01–0.1
a_{ii}	Intraspecific interference coefficient	Arbitrary, larger values allow larger S at IEE
$\sigma^2_{\varepsilon,i}$	Variance of the Gaussian environmental stochasticity parameter ε_e affecting species i	(0 – 0.5)
p_c	$\in (0,1)$, per-time-step probability of immigration	0–0.05
p_{ij}	$\in (0,1)$, probability of edge (link) establishment of the ith reintroduced species with each of the $n – 1$ others	0.05–0.3
δ	$\in (0,1)$, proportion decrease in population size during an extreme event	0.05–0.5
p_δ	$\in (0,1)$, per-time-step probability that an extreme event occurs	0.05–1

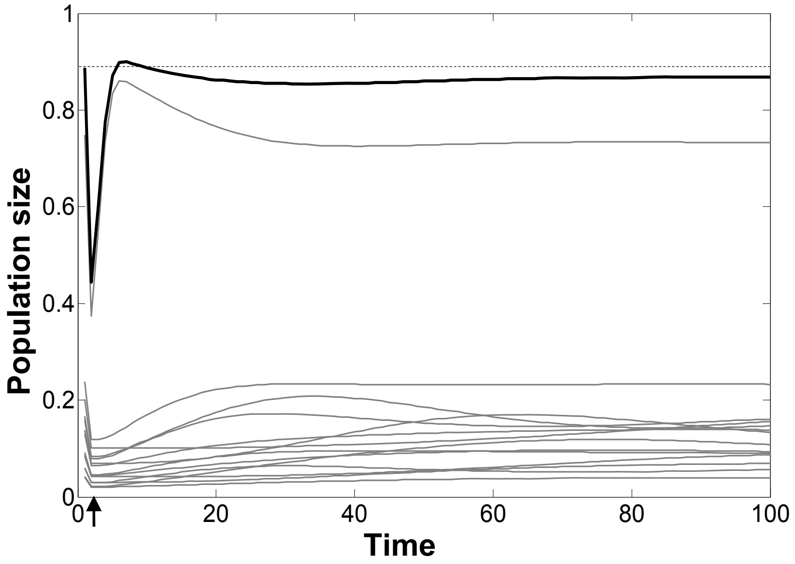

Figure S1. Illustration of resilience in a model food web consisting of 30 species. An environmental fluctuation (point in time indicated by an arrow) decreases population size by 50%, with all populations experiencing the perturbation in a perfectly correlated way (no mismatches) in this example. The thick dark curve is the Euclidean norm of the population vector $||\mathbf{x}||$, while the thinner grey lines show the corresponding population trajectories of the 15 most abundant species in the web (basal species being the most abundant). The norm at equilibrium is shown by the dotted line.

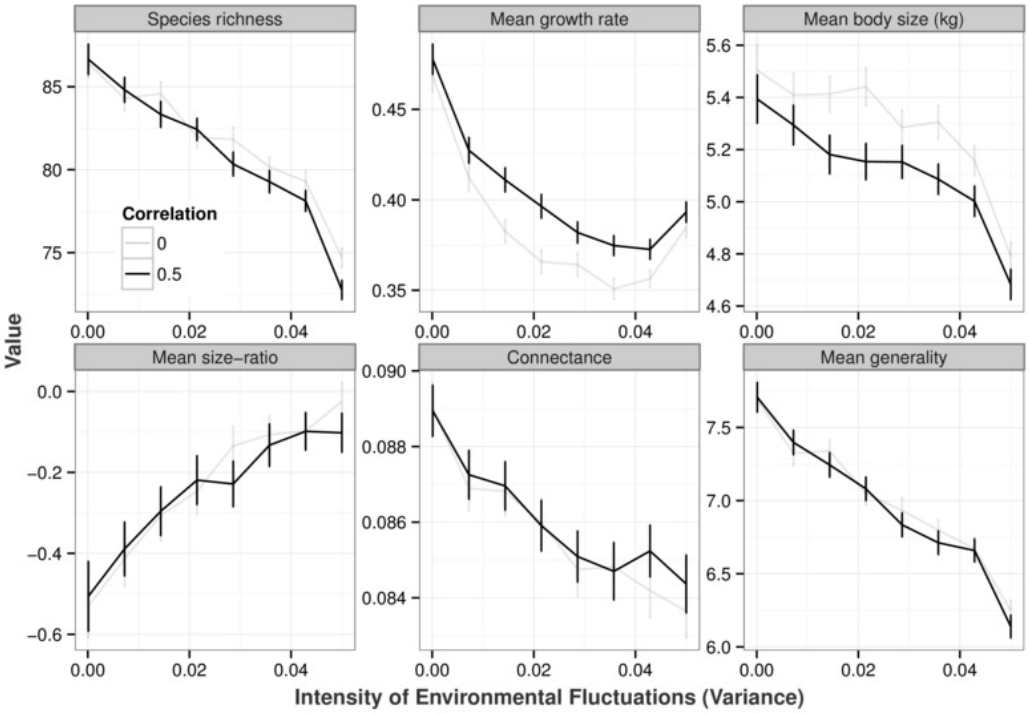

Figure S2: Effects of mismatches between species on the consequences of environmental fluctuations on open, dynamically assembling food webs. Mismatch-level is defined as inverse of cross-correlation between species' sensitivity to fluctuations – so a correlation of 0 implies perfect mismatch between species across the whole community, while a correlation of 1 implies a perfect match such that all species are identically sensitive to fluctuations. A correlation of 1 (not shown) results in qualitatively similar results as those shown above. The bars represent 95% confidence intervals around the mean of 200 community assembly simulations. Each model community at the end of a simulation is at immigration-extinction equilibrium. Note that some food web features are significantly sensitive to mismatches, while others are not.

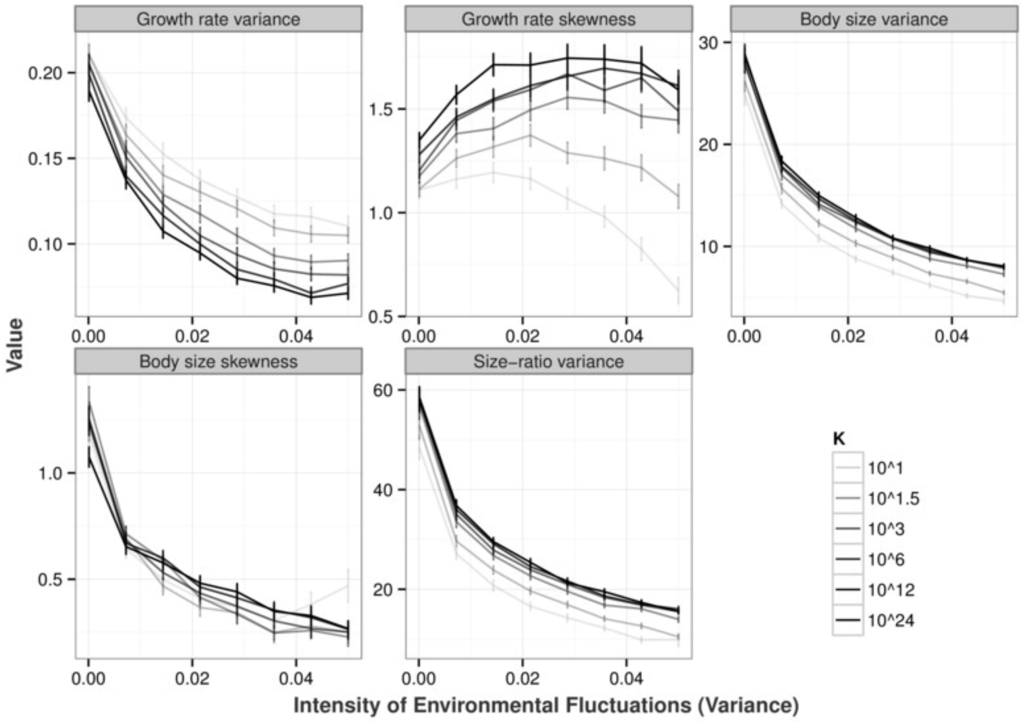

Figure S3: Effects of environmental fluctuations on additional food web features related to body size and growth rates emerging in model communities at immigration-extinction equilibrium.

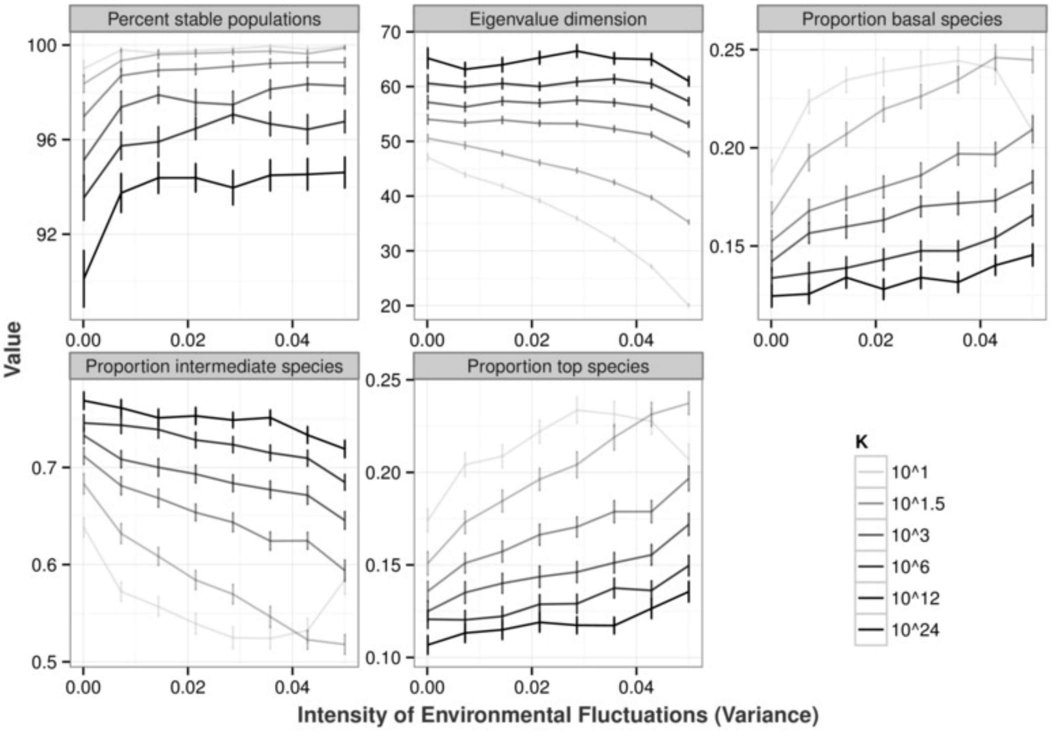

Figure S4: Effects of environmental fluctuations on additional food web features related to food web stability and topology emerging in model communities at immigration-extinction equilibrium.

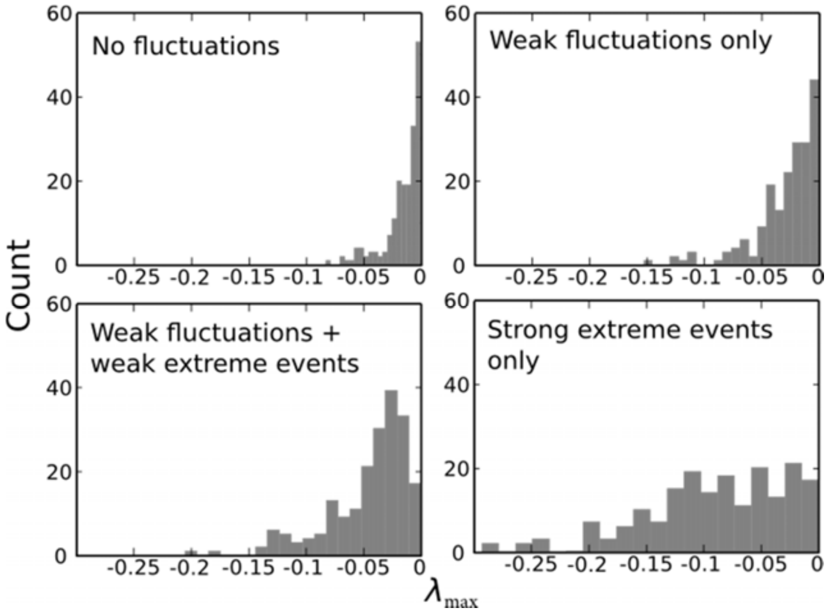

Figure S5: Distribution of λ_{max} (maximum eigenvalue) for each of 200 assembled model food-webs under no stochastic environmental fluctuations or extreme events ($\sigma_e^2 = 0, p_\delta = 0$), weak stochastic environmental fluctuations ($\sigma_e^2 = 10^{-4}, p_\delta = 0$), weak stochastic fluctuations combined with weak and intermittent extreme events ($\sigma_e^2 = 10^{-4}, \delta = 0.1, p_\delta = 0.2$) and (**E**) no stochastic fluctuations but strong and frequent extreme events ($\delta = .1, p_\delta = 1$). Note that for all cases $|\lambda_{max}| \ll \sigma_e^2$ as expected from May's [6] analytical results. $1/|\lambda_{max}|$ of a locally stable dynamical system captures the return time of the system to the equilibrium following perturbation, and is a measure of resilience. All the eigenvalues are negative because we only considered the locally-stable subset of the community — as such, all stochastically assembled communities have at least one species that is on a path to extinction. That is, no dynamically assembling system is locally stable, a result of the constant and inherent immigration-extinction dynamics of open food webs in dynamically changing environments such as running waters.
